# mGem: A quarter century with the Pirofski–Casadevall damage response framework—a dynamic construct for understanding microbial pathogenesis

**DOI:** 10.1128/mbio.02945-24

**Published:** 2025-02-11

**Authors:** Joshua D. Nosanchuk

**Affiliations:** 1 Department of Medicine (Division of Infectious Diseases), Albert Einstein College of Medicine, New York, USA; 2 Department of Microbiology and Immunology, Albert Einstein College of Medicine, New York, USA; Instituto Carlos Chagas, Curitiba, Brazil

**Keywords:** microbial pathogenesis, scientific framework, education

## Abstract

A quarter of a century ago, Liise-anne Pirofski and Arturo Casadevall shared their concepts of microbial pathogenesis through the lens of a damage-response framework (DRF), which characterizes disease by assessing the dynamic interactions between the host and pathogen as reflected by damage as the readout. This framework has evolved to be a powerful tool for understanding the biology of complex infectious diseases, analyzing emerging and reemerging microbes, and developing therapeutic approaches to combat infections. The DRF is also frequently used to explain research at scientific meetings and to teach microbial pathogenesis to diverse learners. This mGem reviews how the DRF came to be and provides an overview of how it is used. Without a doubt, the scientific community will continue to leverage the DRF to advance research and innovate therapeutic approaches, which is especially important as new and reemerging infectious diseases threaten global health.

## PERSPECTIVE

Over 150 years ago, Louis Pasteur (1822–1895) and Robert Koch (1843–1910) battled over fundamental approaches to science stemming from their work on *Bacillus anthracis* with Koch identifying the microbe as the cause of anthrax (1) and Pasteur attenuating the bacterium for vaccination purposes (2). The groundbreaking work of these remarkable investigators resulted in the founding of the fields of immunology (Pasteur) and medical bacteriology (Koch). However, the consequences of their establishing scholarly programs focused exclusively on either the host or pathogen became incompatible with addressing observations that were increasingly occurring in the late 20th century science and medicine.

For example, starting in the 1970s, *Candida albicans*, a common well-known commensal dimorphic fungus, surprisingly and increasingly became a major threat to diverse individuals in whom the fungus caused serious diseases ranging from complicated mucocutaneous infections to life-threatening sepsis. The rapid emergence of the *C. albicans* threat was multi-factorial and due to the widespread introduction of intravenous catheters, particularly large central lines, the use of chemotherapies and steroids, and widespread administration of antimicrobials, and accelerated by the HIV pandemic. Unfortunately, we have not resolved the challenges of *C. albicans*, and the fungus is *now* viewed as a critical global health threat (Fungal Priority Pathogens List [3]). As well-known commensals, such as *Candida*, became associated with a variety of clinical presentations ranging from no disease to life-threatening sepsis, Koch’s four postulates to determine whether a microbe can cause a specific disease, which were explicitly defined by Friedrich Loeffler in 1884 (4), were less applicable. *C. albicans* escapes the fundamental criterion of the postulates—that a microbe should be present in a diseased host but absent in a healthy host. Moreover, according to prior doctrine, a microbe should not be capable of flexing between existing as a commensal or a lethal pathogen/opportunistic pathogen depending on the host’s immune status. On the other hand, a toxin, like botulinum toxin, can be lethal in a diverse range of hosts from immunologically “impaired” individuals to those with “normal” immune systems, demonstrating that the host variable is not always critical to outcomes of host–pathogen interactions.

The identification of the immune reconstitution inflammatory syndrome (IRIS) occurring after different infectious diseases further complicated our understanding of host–pathogen interactions. IRIS was first characterized in the 1980s in patients with mycobacterial diseases—both tuberculosis and leprosy—who developed a constellation of findings including fever, shortness of breath, fatigue, and weight loss in response to antimicrobial therapy (5), and individuals with leprosy also had worsening of skin lesions (6). The incidence of IRIS markedly increased with the introduction of antiretrovirals highly active against HIV, especially following the addition of protease inhibitors in 1996, particularly in settings of co-infections with tuberculosis, cryptococcosis, and pneumocystis, which have been linked to factors including microbial antigen concentration, host genetics, and host immunological state (7). These complex interactions did not fit with prior pathogenesis models.

In response to these newly realized phenomena and evolving disease manifestations in response to treatments, scientists and physicians struggled to address the new microbial pathogenesis environment. In her seminal 1994 paper “Tolerance, danger and the extended family,” Polly Matzinger at the National Institutes of Health (NIH) strikingly wrote that she “abandoned this belief” “that the immune system’s primary driving force is the need to discriminate between self and non-self” (8). Instead, she thoughtfully posited that the immune system “receives positive and negative communications from an extended network of other bodily tissues.” Matzinger’s prescient position set the stage for new views of mechanisms for microbial pathogenesis.

Hence, there was a massive gap at the end of the last century as to how to critically and effectively understand microbial pathogenesis, which made teaching the topic rather flummoxing. As co-directors of a course on microbial pathogenesis at Albert Einstein College of Medicine (Einstein), Liise-anne Pirofski and Arturo Casadevall created an innovative structure, the damage response framework (DRF), to address these challenges by including dynamic variables of host and pathogen interaction. They first piloted the novel construct in their course and, then, formally wove their framework into both Einstein’s PhD and MD programs’ microbiology, immunology, and infectious diseases curricula. They concomitantly curated their new approach to host–pathogen interactions into a manuscript, and their seminal work entitled “Host-pathogen interactions: redefining the basic concepts of virulence and pathogenicity” that described the DRF was published in the American Society for Microbiology’s journal *Infection and Immunity* in August 1999 (9).

**Fig 1:**
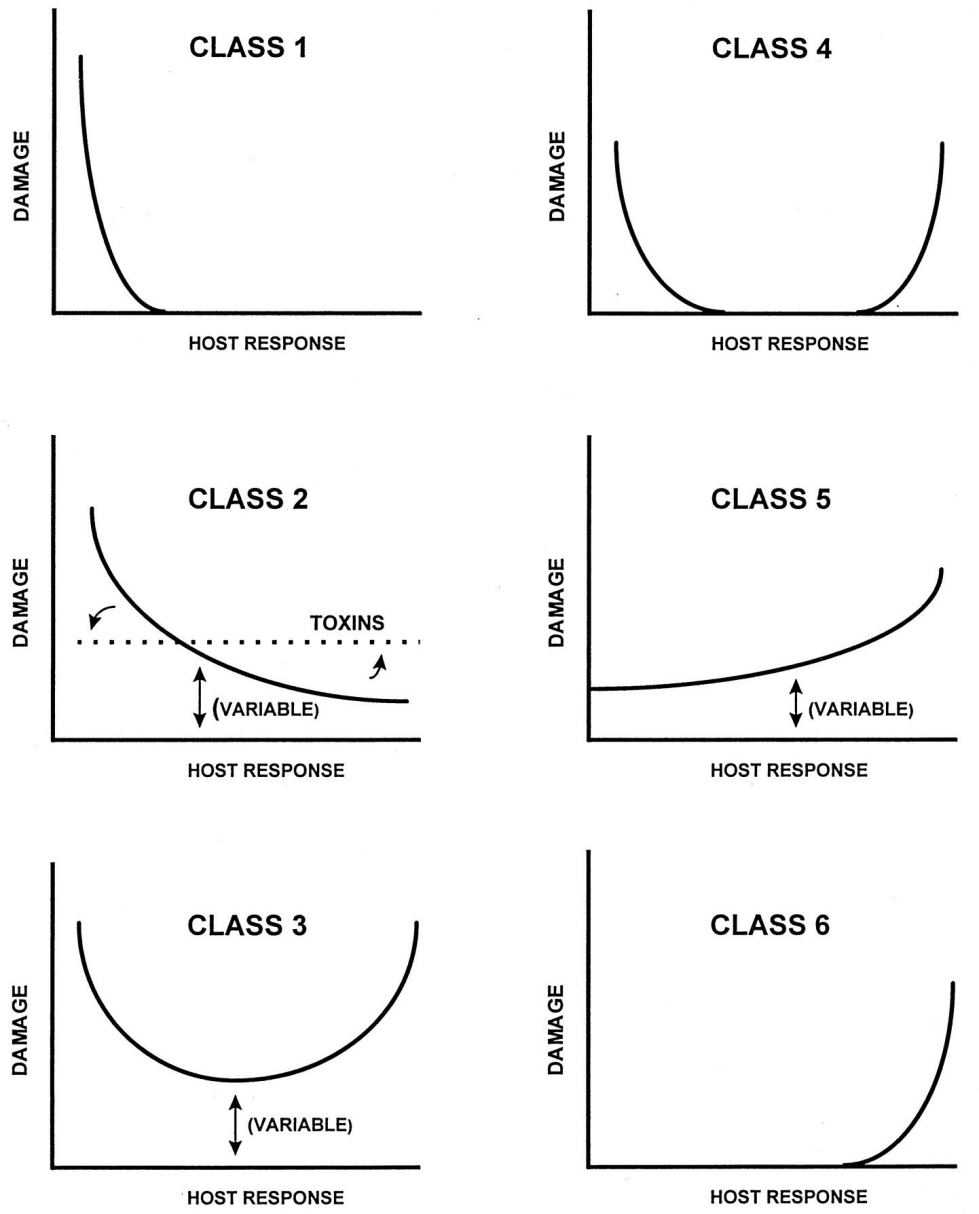
The original six classes of damage–response curves comprising the Pirofski–Casadevall DRF (9). The amount of host damage is shown on the *Y* axis with the strength of the associated host response presented on the *X* axis. A variable is present in classes 2, 3, and 5 to represent a range in damage given differences in individual hosts. Reproduced from reference 9 with permission.

The genius in Pirofski and Casadevall’s DRF (9) was twofold: (i) adding the variable of the interaction between microbe and host together with facets of the individual microbe and host and (ii) focusing on host damage as the readout for disease. Significantly, host damage may be the direct outcome of microbial processes or due to the response of the host to the microbe or its products. Figure 1 from their 1999 work demonstrates the critical role of host damage as the readout for the interaction. Although Pirofski and Casadevall continue to enhance the DRF as new insights arise (10), the concepts presented remain fundamentally as detailed in the 1999 framework. For example, class 1 microbes lead to damage only in the setting of a weak host response and include, for example, *Pneumocystis jirovecii* and *Legionella pneumophila*. In contrast, *Helicobacter pylori* causes gastritis and malignancies only in the setting of a robust host response, a class 6 microbe. However, *H. pylori* is an example of the “living document” approach to the DRF as the presence of the microbe has since been found to be inversely associated with (e.g., protective against) Barrett’s esophagus (11, 12), and Pirofski and Casadevall subsequently modified the class 6 curve to extend below the *X* axis to demonstrate this beneficial response in the setting of a less robust immune recognition of the microbe (10).

**Fig 2:**
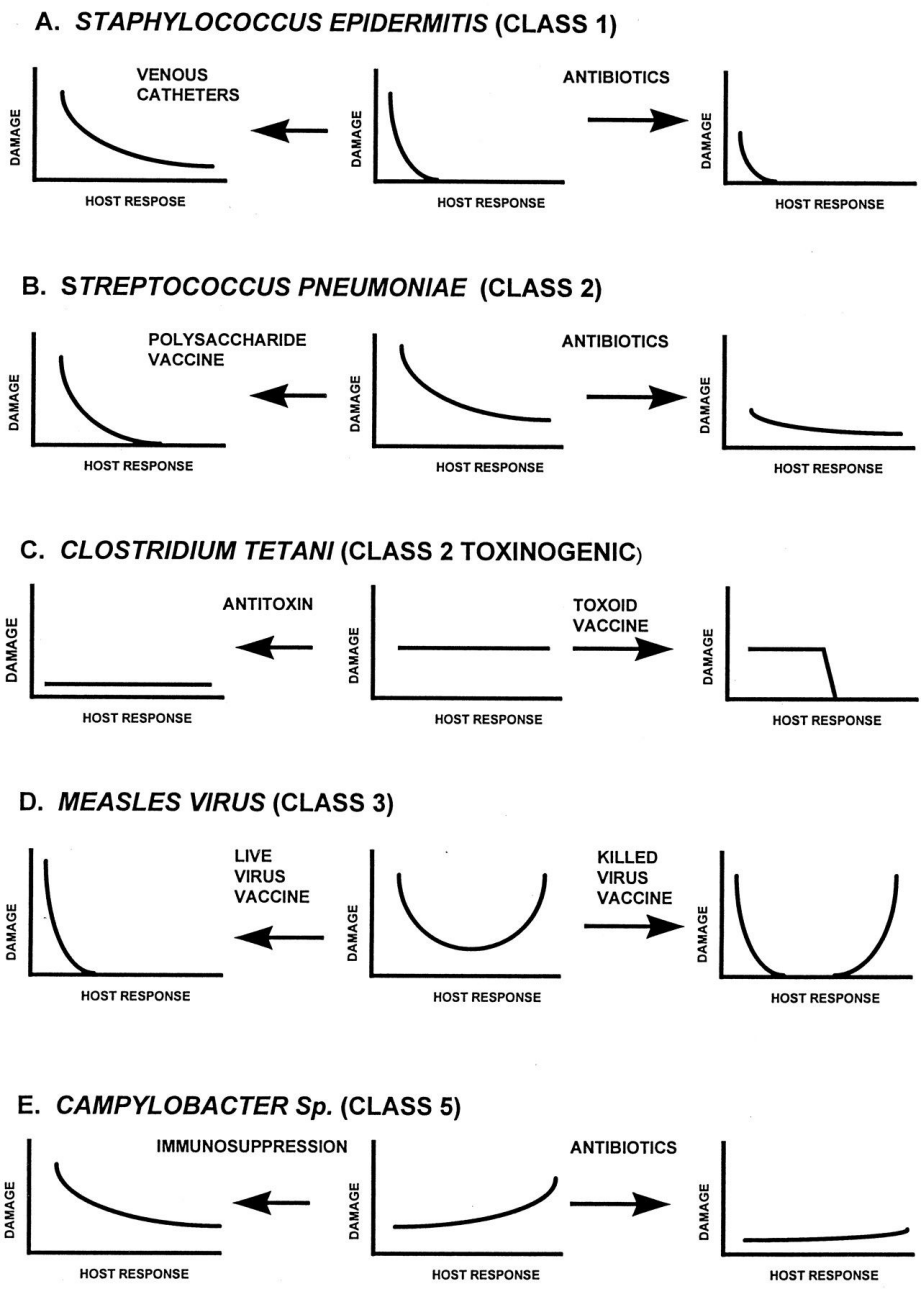
Exemplars of how therapeutic interventions can markedly shift damage–response curves in the Pirofski–Casadevall DRF (9). Reproduced from reference 9 with permission.

Another groundbreaking aspect of the DRF was that the framework effectively shows how therapeutic interventions can positively or negatively impact the damage–response curves. Figure 2 from the 1999 paper (9) demonstrates how catheters, antibiotics, immunosuppressants, vaccines, and antibodies shift outcomes for several clinically important infectious diseases. The DRF allows for dynamic shifts in the microbe–host interplay. This is incredibly important as we continue to modify host immune response with novel biologics, as many of these can lead to unexpected susceptibilities to serious infectious diseases (13). The DRF is also dynamic as pathogens adapt and can manipulate host interactions. Moreover, the DRF is a powerful tool for scientists and physician–investigators to understand the pathogenesis of infectious diseases (14).

Numerous publications have leveraged the DRF to study diverse infectious diseases. The conundrum of *Candida* described above is thoughtfully addressed by the Fidel and Noverr groups through the DRF, and the framework is used to present approaches to mitigate disease through modification of host damage responses (15, 16), which is consistent with the original thoughts outlined in the 1999 DRF paper (9). Peter Williamson at NIH applied the DRF to critically evaluate major central nervous system infections, including pneumococcus, malaria, tuberculosis, and cryptococcus, and to demonstrate how distinct clinical approaches for each can lead to better outcomes (17). Importantly, the DRF is applicable to new or emerging diseases and can lead to the implementation of therapeutic approaches to combat these novel infections. In July 2020, Pirofski and Casadevall deployed the DRF to elucidate the pathogenesis of coronavirus disease 2019 (COVID-19) (18). The DRF was subsequently applied by additional groups to enhance and innovate the treatment of COVID-19 (19, 20).

The DRF has been proposed as a tool to assess individual patient risks for developing an infectious disease. A multi-institutional group demonstrated that information on microbiome, immunity, sex, temperature, environment, age, chance, history, inoculum, nutrition, and genetics (MISTEACHING) could be analyzed with machine learning to generate data to form patient-specific infection control interventions (21). Although some electronic medical record systems and other algorithms currently utilize some of these data to, for example, trigger alerts for concerns for sepsis, this collective information has yet to be leveraged for patient care, but the potential remains enormous for impactful interventions.

Of enormous impact, the DRF has been globally adopted for teaching microbial pathogenesis. The DRF continues to be utilized in both PhD and MD programs at Einstein. For example, Attila Gacser uses the DRF to teach to diverse students in Szeged, Hungary, and Marcio Rodrigues incorporates the framework into his teaching in Curitiba and Rio de Janeiro, Brazil (personal communications). Similarly, Sirida Youngchim in Chiang Mai, Thailand, lectures using the DRF, and earlier this year, we collaboratively published an assessment of the virulence of *Talaromyces marneffei*, a World Health Organization-identified priority pathogen, through the lens of the DRF (22). Furthermore, I have personally seen the DRF used several dozen times to explain people’s research in diverse international meetings (e.g., ASM Microbe, IDWeek, ESCMID Global, Gordon Research Conferences, etc.). Also, the framework regularly pops up on social media platforms, such as Twitter (also known as “X”), in images of microbial pathogenesis investigators presenting their work. The regular use of the DRF among diverse scientific and medical communities underscores the massive impact of the framework for both advancing discovery and explaining complex concepts in microbial pathogenesis.

In the past quarter century, with the Pirofski–Casadevall DRF, the framework has become a major force in science and medicine. The framework has directly impacted how we analyze infectious diseases, and it has helped us formulate innovative approaches to combat infections in diverse populations and individual patients, as highlighted by the early call for immune therapies in April 2020 (23) when the COVID-19 pandemic was running hot in the Bronx and rapidly spreading globally. The DRF will have an enduring impact as, to date, it has been used to educate literally tens of thousands of students about microbial pathogenesis, influencing how our next generation of scientists, clinicians, public health practitioners, and others view microbes and hosts. In 2020, Anthony Fauci titled a paper “It ain't over till it’s over…but it’s never over—emerging and reemerging infectious diseases” highlighting that vigilance is required as we will certainly continue to combat new infectious challenges (24). Fortunately, we have the Piroski–Casadevall framework to thoughtfully confront these new clinical conundrums.
